# Thyrotoxic Periodic Paralysis Manifesting as Acute Respiratory Failure: A Case Report

**DOI:** 10.7759/cureus.105388

**Published:** 2026-03-17

**Authors:** Daniel Balderrama, Samhara A Espinosa, Ana M Barboza

**Affiliations:** 1 Internal Medicine, Programa Multicentrico de Especialidades Médicas del Tecnológico de Estudios Superiores de Monterrey, Monterrey, MEX; 2 Geriatrics, Programa Multicentrico de Especialidades Médicas del Tecnológico de Estudios Superiores de Monterrey, Monterrey, MEX

**Keywords:** acute flaccid paralysis, acute quadriparesis, clinical case report, hypokalemia, hypoxic respiratory failure, quadriparesis with areflexia, thyrotoxicosis, thyrotoxic period paralysis

## Abstract

Thyrotoxic periodic paralysis is an uncommon endocrine emergency marked by sudden muscle weakness linked to hypokalemia in the context of thyrotoxicosis. Cases have increasingly been reported worldwide, even though they are more common in Asian populations. Early detection is crucial because of the risk of life-threatening complications such as arrhythmias and respiratory failure.

We present the case of a 42-year-old man with no significant medical history who exhibited sudden palpitations, diaphoresis, and anxiety, subsequently developing rapidly progressive lower extremity weakness, culminating in flaccid quadriparesis and acute respiratory failure requiring endotracheal intubation. Neurological examination indicated generalized weakness (0/5 strength) and areflexia, with no sensory deficit or cranial nerve involvement. Laboratory tests showed severe hypokalemia (1.3 mEq/L) and biochemical thyrotoxicosis, with low thyroid-stimulating hormone (TSH) levels (0.01 μIU/mL) and high free thyroxine (T4) levels (3.07 ng/dL). Neuroimaging and cerebrospinal fluid analysis yielded normal results. After carefully replacing potassium, the patient showed rapid clinical improvement with complete neurological recovery. Respiratory failure requiring mechanical ventilation is an uncommon presentation of thyrotoxic periodic paralysis and highlights the importance of early recognition.

Thyrotoxic periodic paralysis must be contemplated in patients exhibiting acute flaccid paralysis and significant hypokalemia. Prompt diagnosis and treatment are crucial to prevent potentially fatal complications, such as respiratory failure or cardiac arrhythmias, which can arise from untreated thyrotoxic periodic paralysis.

## Introduction

Thyrotoxic periodic paralysis is an uncommon complication of hyperthyroidism, marked by intermittent muscle weakness linked to hypokalemia [1]. Thyroid hormones and adrenergic activity stimulate heightened sodium (Na+)/potassium (K+) ATPase activity, which facilitates intracellular potassium redistribution and causes the condition [2-4].

Although thyrotoxic periodic paralysis has been predominantly reported in Asian populations, where the prevalence among patients with hyperthyroidism is estimated to be approximately 1.8%-1.9%, cases have increasingly been described in other ethnic groups [2,5-7]. Higher-than-expected prevalence has also been reported in Hispanic and Polynesian populations, whereas the condition remains relatively uncommon in Caucasian and Black populations, with estimated prevalence rates of approximately 0.1%-0.2% among patients with hyperthyroidism [5-7]. Clinical manifestations vary from proximal muscle weakness [3,5] to profound paralysis, potentially affecting respiratory muscles [2,8].

Early recognition is crucial, as potassium replacement therapy facilitates swift clinical recovery and mitigates life-threatening complications [1].

## Case presentation

A 42-year-old man with no notable medical history arrived at the emergency department, exhibiting a 12-hour history of palpitations, diaphoresis, and anxiety. Over the following hours, he developed progressive weakness in the lower extremities, which rapidly worsened and progressed to generalized flaccid paralysis prior to admission.

Shortly after being admitted, the patient developed acute respiratory failure that needed endotracheal intubation and mechanical ventilation. The physical examination revealed alert mental status, no cranial nerve abnormalities, no sensory deficits, generalized muscle weakness (0/5 strength), generalized areflexia, tachycardia, and tachypnea.

Laboratory findings on admission are summarized in Table 1. Biochemical analyses were performed in the hospital laboratory using standard automated laboratory methods, including ion-selective electrode analysis for electrolytes and chemiluminescent immunoassays for thyroid function tests. The laboratory evaluation revealed severe hypokalemia (1.3 mEq/L; reference range: 3.5-5.0 mEq/L) and biochemical thyrotoxicosis with suppressed thyroid-stimulating hormone (TSH) (0.01 μIU/mL; reference range: 0.4-4.0 μIU/mL) and elevated free thyroxine (T4) (3.07 ng/dL; reference range: 0.8-1.8 ng/dL). The most clinically significant finding was the profound hypokalemia, which explained the rapid development of flaccid paralysis and respiratory failure. Cerebrospinal fluid analysis showed normal findings, including the absence of pleocytosis and normal protein levels.

**Table 1:** Laboratory findings on admission. TSH, thyroid-stimulating hormone; T4, thyroxine; T3, triiodothyronine; CSF, cerebrospinal fluid; LDH, lactate dehydrogenase

Laboratory test	Result	Reference range
Potassium	1.3 mEq/L	3.5-5.0 mEq/L
Chloride	110 mEq/L	98-107 mEq/L
TSH	0.01 μIU/mL	0.4-4.0 μIU/mL
Free T4	3.07 ng/dL	0.8-1.8 ng/dL
T3	1.69 ng/mL	0.8-2.0 ng/mL
CSF pH	8.0	7.28-7.32
CSF glucose	92 mg/dL	50-80 mg/dL
CSF protein	0.24 g/L	0.15-0.45 g/L
CSF LDH	14 U/L	<40 U/L
CSF cell count	0 cells/mm³	0-5 cells/mm³

**Figure 1:**
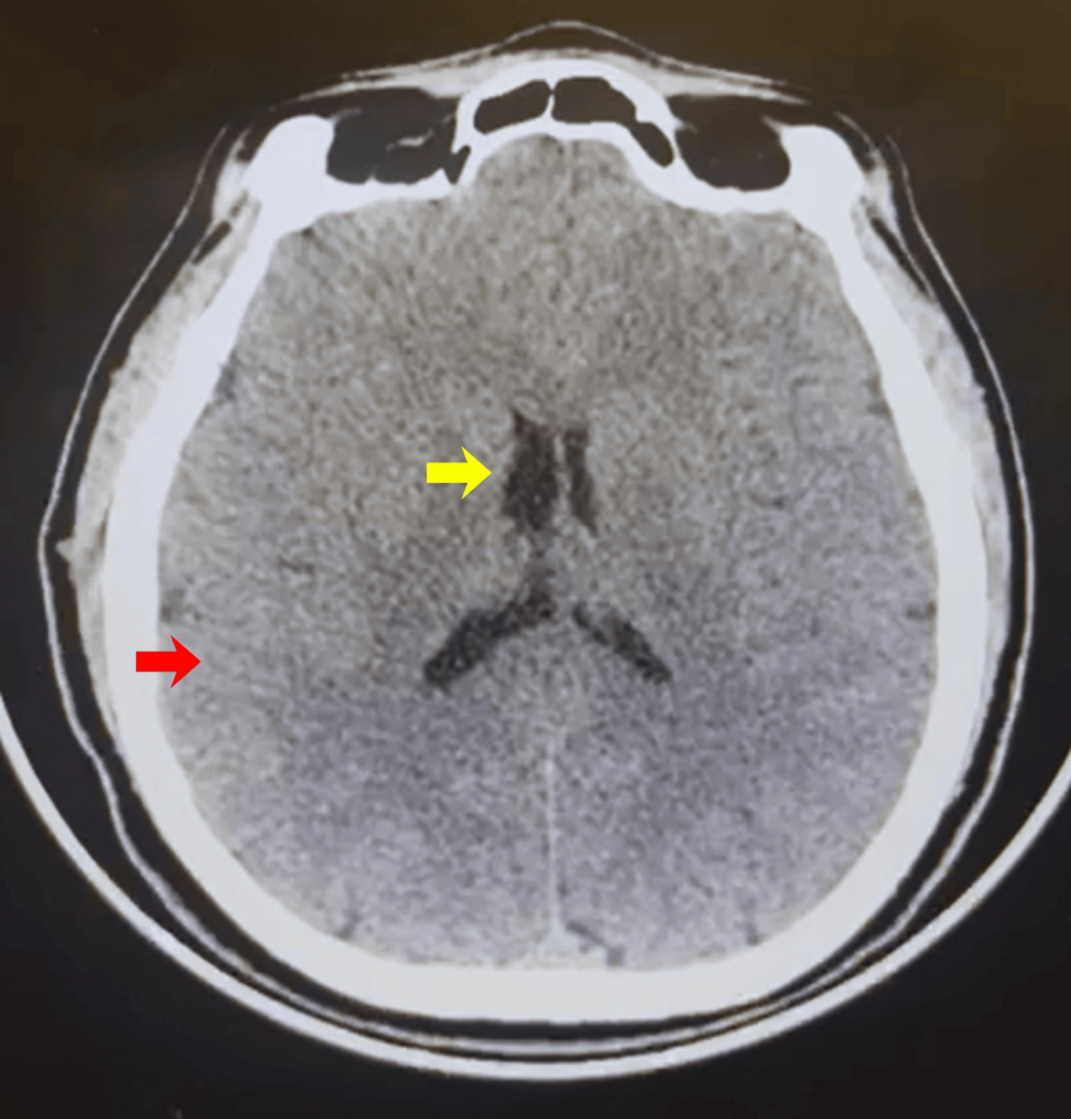
Axial non-contrast brain computed tomography. Axial non-contrast brain computed tomography demonstrating preserved gray-white matter differentiation and no evidence of intracranial hemorrhage, mass effect, or acute ischemic changes. The yellow arrow indicates the lateral ventricle, and the red arrow indicates preserved gray-white matter differentiation.

Brain computed tomography demonstrated preserved gray-white matter differentiation with no evidence of intracranial hemorrhage, mass effect, or ischemic changes (Figure 1). Neuroimaging is frequently performed to exclude structural causes of acute paralysis. Thyroid ultrasonography could not be performed due to a lack of availability.

The patient received intravenous potassium replacement, with an estimated total replacement of approximately 60 mEq over the first 12 hours under continuous cardiac monitoring, given the risk of rebound hyperkalemia in thyrotoxic periodic paralysis. After stabilization, treatment for thyrotoxicosis was initiated with the nonselective beta-blocker propranolol (40 mg every eight hours) and the antithyroid drug methimazole (20 mg daily), and the same regimen was continued at discharge. Clinical improvement occurred rapidly with the progressive recovery of muscle strength. The patient was successfully extubated without complications and discharged with complete neurological recovery.

## Discussion

Thyrotoxic periodic paralysis is a rare but possibly fatal complication of thyrotoxicosis. The underlying mechanism involves the increased activity of the Na+/K+ ATPase pump stimulated by thyroid hormones, catecholamines, and insulin, leading to intracellular potassium shift and transient hypokalemia [2,5]. It is marked by sudden episodes of muscle weakness caused by a shift of potassium within cells rather than true total body potassium depletion.

Episodes are often triggered by factors such as meals high in carbohydrates, intense exercise, alcohol consumption, or stress. In certain instances, a definitive trigger remains unidentified [2,9].

Most patients present with proximal muscle weakness in their lower limbs, but in severe cases, their condition can lead to quadriparesis and respiratory failure that needs ventilatory support. Several conditions can present with acute flaccid paralysis, making the early recognition of thyrotoxic periodic paralysis essential for appropriate management. Similar clinical manifestations have been described in other forms of periodic paralysis affecting skeletal muscle excitability [3,4,10]. The differential diagnosis of acute flaccid paralysis with hypokalemia is summarized in Table 2 [2,8]. Treatment involves careful potassium replacement and the definitive management of hyperthyroidism to prevent recurrence [2,6].

**Table 2:** Differential diagnosis of acute flaccid paralysis with hypokalemia. Adapted from previously published literature [2,8]. TSH: thyroid-stimulating hormone

Condition	Key clinical features	Laboratory findings	Distinguishing features
Thyrotoxic periodic paralysis	Acute proximal muscle weakness, often in the lower extremities	Severe hypokalemia with suppressed TSH and elevated thyroid hormones	Reversible paralysis after potassium correction and the treatment of thyrotoxicosis
Familial hypokalemic periodic paralysis	Recurrent episodes of weakness often beginning in adolescence	Hypokalemia during attacks	Positive family history and normal thyroid function
Guillain-Barré syndrome	Progressive ascending weakness with areflexia	Cerebrospinal fluid with albuminocytologic dissociation	Often associated with preceding infection and sensory symptoms
Hypokalemia due to renal or gastrointestinal loss	Muscle weakness with systemic symptoms	Hypokalemia with metabolic alkalosis or gastrointestinal losses	History of vomiting, diarrhea, or diuretic use

Prior studies have shown that thyrotoxic periodic paralysis occurs predominantly in Asian populations, with an estimated incidence of approximately 1.8%-1.9% among patients with thyrotoxicosis and a marked male predominance. More recent data have provided further insight into its epidemiology. A population-based registry study from Hong Kong reported that approximately 1.2% of patients with thyrotoxicosis developed thyrotoxic periodic paralysis, also with a clear predominance in men. Interestingly, although the incidence of thyrotoxicosis increased over the past two decades, the incidence of thyrotoxic periodic paralysis remained relatively stable [8,11]. This observation suggests that additional factors, including environmental or lifestyle influences, may contribute to its development. Most cases described in the literature occur in young men, and in some patients, thyrotoxic periodic paralysis may be the first manifestation of hyperthyroidism. Although most patients present with limb weakness, severe cases with respiratory muscle involvement and acute respiratory failure have also been reported, similar to the presentation observed in our patient [12,13].

## Conclusions

Thyrotoxic periodic paralysis must be included in the differential diagnosis of acute flaccid paralysis linked to hypokalemia, especially in patients exhibiting symptoms of thyrotoxicosis.

Early recognition is crucial as the condition is reversible with timely potassium correction and the suitable management of the underlying thyroid disorder. If this condition is not recognized, it could lead to severe complications such as life-threatening arrhythmias or respiratory failure. A quick diagnosis and treatment can speed up recovery and prevent recurrent episodes.
